# The Modern Environment: The New Secondary Cause of Hypertension?

**DOI:** 10.3390/medicina59122095

**Published:** 2023-11-29

**Authors:** Konstantinos Rossios, Christina Antza, Vasileios Kachtsidis, Vasilios Kotsis

**Affiliations:** 1 Cardiology Clinic, Papageorgiou Hospital, Aristotle University of Thessaloniki, 54124 Thessaloniki, Greece; kostasrossios@yahoo.gr; 2 Hypertension Center, 3rd Department of Medicine, Papageorgiou Hospital, Aristotle University of Thessaloniki, 54124 Thessaloniki, Greece; kris-antza@hotmail.com (C.A.); billykachtsidis@gmail.com (V.K.)

**Keywords:** hypertension, environment, noise pollution, seasonal variability, temperature, obesity

## Abstract

The most important risk factor for cardiovascular disease, the leading cause of death worldwide, is hypertension. Although most cases of hypertension are thought to be essential, the multifactorial associations of the environmental influence on blood pressure seem to play an important role and should be more closely investigated. This review attempts to focus on the recent literature that examines the environmental effects on arterial blood pressure and its management. Seasonal variability and the role of ambient temperature, either occupational or recreational noise pollution, as well as obesity due to environment-caused dietary habits, are recognized as important risk factors, affecting the onset as well as the regulation of hypertension. Furthermore, the effects of seasonal fluctuations in blood pressure, noise pollution, and obesity seem to share a similar pathogenesis, and as such to all further react together, leading to increased blood pressure. The activation of the autonomous nervous system plays a key role and causes an increase in stress hormones that generates oxidative stress on the vascular system and, thus, vasoconstriction. In this review, by focusing on the association of the environmental impact with arterial blood pressure, we come to the question of whether most cases of hypertension—if not all—should, indeed, be considered primary or secondary.

## 1. Introduction

Cardiovascular disease (CVD) is the top cause of death worldwide and, despite the scientific attempts, hypertension still remains one of the main cardiovascular risk factors [1]. According to the World Health Organization, 1.13 billion people worldwide have hypertension, and the numbers are expected to become higher in the following years [2]. In the United States, nearly half of adults (47%) have hypertension, defined either as abnormal values of blood pressure (BP) or as the use of antihypertension treatment [3]. In Europe, the prevalence is lower but still remarkable, coming close to 25% [4]. The control rates of the hypertensive population seem also to be dramatic; the condition of 25% of that population is reported to be uncontrolled and almost half of them live with BP values higher than 140/90 mmHg [3]. According to the May Measurement Month 2017, which is an annual initiative launched by the International Society of Hypertension as a way to tackle the widespread lack of awareness regarding hypertension, more than 1.2 million individuals from 80 different countries were enrolled and subjected to analysis, with nearly one-third of them being identified as hypertensive. Among this hypertensive group, there were over 100,000 individuals not receiving treatment for high BP, and more than 150,000 individuals who were undergoing hypertension treatment but still had inadequate control of BP [5]. In the attempt to confine CVD, it is of utmost importance to restrain the large prevalence of hypertension and, as so, to investigate other causes that negatively affect BP.

Hypertension, a medical condition characterized by systolic BP values equal to or exceeding 140 mmHg and/or diastolic BP values equal to or exceeding 90 mmHg, has been rigorously defined through extensive research, drawing upon evidence gathered from numerous randomized controlled trials. This body of research unequivocally demonstrates the substantial benefits derived from treating patients whose BP falls within or surpasses these specified values when managed in a clinical setting [6]. Blood pressure results from the interplay of cardiac output and systemic vascular resistance. Consequently, individuals suffering from arterial hypertension can experience elevated cardiac output, increased systemic vascular resistance, or a combination of both factors. Various growth factors, such as angiotensin and endothelins, induce an increase in vascular smooth muscle mass, a process known as vascular remodeling. The renin–angiotensin–aldosterone system significantly contributes to the regulation of arterial pressure and sodium balance within the body. Substantial evidence suggests that the resetting of pressure natriuresis significantly contributes to the onset of hypertension [7]. Numerous established factors contribute to the development of hypertension, including primary aldosteronism, fibromuscular dysplasia, atherosclerosis, pheochromocytoma, and obstructive sleep apnea [8].

An acknowledgement of novel elements—further than the well-known, previously reported ones—that influence the development and management of hypertension seems, nowadays, to be more than crucial. Evidence suggests that environmental factors play a key role not only in the onset of hypertension but also in the success of the treatment. Specifically, components like climate change, air pollution, traffic noise, and socioeconomic status seem to have direct effects that lead to high BP [9]. Taking into account the great incidence of hypertension as well as the existence of uncontrolled cases [3] despite the large effort from the scientific community, we have to investigate if the environment is the answer to our problem.

Hence, this review aims to study the environmental impact on hypertension by searching the recently published literature regarding seasonal variation, noise pollution, and obesity.

## 2. Seasonal Variation and Hypertension

It is known that hypertension exhibits seasonal variation. BP seems to be higher in the colder months of the year than in the summer [10]. These changes happen through vasoconstriction and a rise in peripheral resistance resulting from exposure to low outdoor temperatures [11]. As such, the activation of the sympathetic nervous system as well as the renin–angiotensin–aldosterone index in the seasonality of hypertension is of major importance [12]. There is, of course, a multi-factorial background to these changes in BP levels. Elements like the restriction in physical activity, the increased food intake, and inadequate home heating can be responsible for these alternations during the winter. Patients well treated for high blood pressure can experience a deregulation during periods of low ambient temperature [13]. Furthermore, this observation agrees with the rise of cardiovascular events during the same time period, bearing in mind that one of the main risk factors for cardiovascular diseases is hypertension [14]. This concerns both men and women of all ages, as well as individuals being treated or not for hypertension.

It has been shown that there is an increase in systolic blood pressure of up to 6.7 mmHg and for diastolic blood pressure of up to 2.1 mmHg, for every 10 °C decrease in outdoor temperature. Additionally, an inverse association between outdoor temperature and cardiovascular events’ morbidity has been described. This association was mediated by an increase in blood pressure [15].

The relationship between hypertension’s seasonality and age was examined in another study based on routine measurements of blood pressure in primary care health system. Older patients (aged 81 or older) presented a greater effect in the elevation of systolic blood pressure regarding the decrease in outdoor temperature than younger patients. No other differences in systolic blood pressure were found when other characteristics like BMI were examined. In general, this study showed that seasonality does exist for all hypertensive patients but was greater in older individuals [16].

Data show that weather changes can even impact the number of hospital admissions for hypertension. Specifically, a decline of 5 °C can lead to a 3% increase in risk for hospital admissions, with short-term changes in temperature shown to not affect the number of admissions, which is opposed to the effects of long-term decreases in temperature. This study was in accordance with other reports, showing that higher temperatures during summertime did not lead to an increase in hospital admissions for hypertension [17,18,19].

Seasonal variability in the onset of essential hypertension was evaluated in another retrospective observational study, not only by office measurements but also by the use of 24 h ambulatory blood pressure measurement in patients admitted for first-diagnosed primary hypertension [20]. The results showed that both systolic and diastolic BP, as well as their drop and their variation during the 24 h recording periods, were all associated with a new onset of essential hypertension in patients admitted during the winter. Furthermore, these patients had higher measurements of blood pressure when compared to patients admitted in other seasons [20].

Hence, controlling the increase in BP during winter is of major importance, and techniques such as changing the indoor temperature or the antihypertensive treatment may help. Indeed, the effects of the adjustment of indoor temperature and antihypertensive medication on the reduction in the winter surge have been studied during the latest years [21]. The rise in indoor temperature by 1 °C could lead to a reduction in systolic BP of up to 0.37 mmHg in the morning and 0.45 mmHg in the evening, as revealed by home monitoring. The effect of outdoor ambient temperature was weaker compared to indoor temperature [22]. The probability of having an abnormal value of BP is lower when the indoor temperature is higher than 12 °C for men >60 years old, 19 °C for men >70 years old, and 24 °C for men >80 years old, while for women the decrease seems to occur at lower temperatures; 11 °C for >70 years old and 16 °C for >80 years old [23]. Moreover, early adjustment of antihypertensive drugs was more efficient in controlling the increase in BP during the colder time of the year. An increase in the dosage of the treatment is preferable from September through November, rather than than later in the year (December–February). Also, patients that started antihypertensive medication in the autumn–winter period had better results compared to when the initiation of treatment took place in spring–summer [24].

Another interesting point was made in a cross-sectional study with more than 4000 participants by Narita et al. that used the database of the J-HOP study about the role of seasonal variation in hypertension in target organ damage [25]. The urine albumin–creatinine ratio (UACR) and serum B-type natriuretic peptide (BNP) level were used to quantify the effect of the increased BP during wintertime on target organs. First of all, it was confirmed that morning home BP was found to be higher in winter, and that this effect was stronger than in the evening home blood pressure and office blood pressure. Furthermore, morning home BP in winter was better correlated with the worsening of renal function and the increase in BNP in the same season. Moreover, a higher incidence of masked hypertension in winter, as well as an increase in white coat hypertension during summer, were observed, and thus, the out-of-office BP monitoring seems to be of major importance during this time of the year.

The seasonal variability in hypertension in patients undergoing hemodialysis was also examined [26]. Consistent with observations regarding changes in BP throughout the year in the general population, this research revealed an elevation in BP during the winter season also in patients undergoing hemodialysis. December, one of the coldest months, exhibited the highest mean pre-dialysis systolic (148 ± 23 mmHg) and diastolic BP (87 ± 17 mmHg), whereas June recorded the lowest pre-dialysis systolic BP (144 ± 23 mmHg) and July the lowest pre-dialysis diastolic BP (85 ± 16 mmHg). These findings align with previous studies of similar interest [27,28,29].

**Figure 1:**
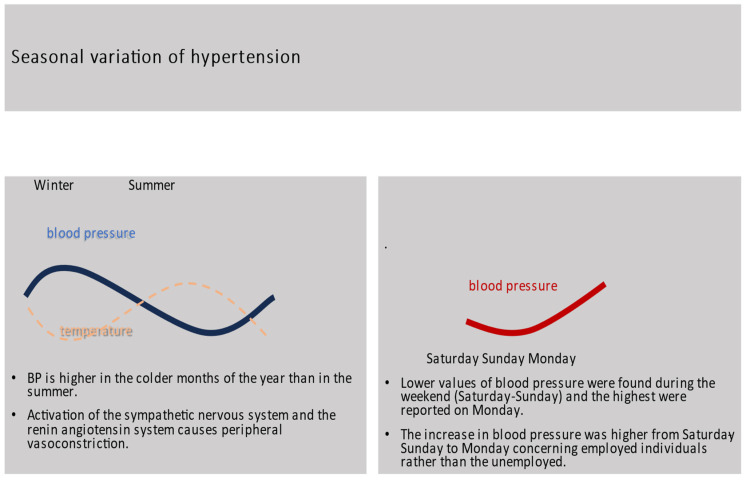
Seasonal variation in hypertension.

The variation in hypertension is not only associated with seasons but also with days of the week. An interesting observation was made in the Finn Home Study that documented the changes in hypertension during the week [30]. Within-subject systolic (128.7 ± 19.2–130.4 ± 19.8) as well as diastolic (79.5 ± 9.8–80.6 ± 9.9 mmHg) BP variation between different days of the week was small but significant. The lower values were found during the weekend (Saturday–Sunday) and the highest were reported on Mondays. This increase in blood pressure was higher from Saturday–Sunday to Monday when considering employed individuals rather than the unemployed [30]. These findings, along with several other reports about the day-by-day variation in hypertension, stress the environmental, socioeconomic, and psychosocial influences on the levels and management of blood pressure. Figure 1 shows the variation in hypertension based on seasons and days of the week.

## 3. Hypertension, Noise, and Air Pollution

There is a strong relationship between noise pollution and cardiovascular events. As claimed by the WHO, 1.6 billion healthy life years are lost every year in Western Europe because of transportation noise [31]. Traffic noise can cause oxidative stress in the vasculature, promoting hypertension and, therefore, increasing the risk of cardiovascular illness. Also, the activation of the hypothalamic–pituitary–adrenal axis, which can cause the release of stress hormones (like cortisol) and the deregulation of glucose adjustment and blood pressure, may offer another possible explanation. Additionally, noise pollution can cause sleep disturbances and alternations in the circadian rhythm leading to the release of stress hormones and endothelial dysfunction, which can result in hypertension [32].

A recently published systematic review and meta-analysis regarding the relationship between occupational noise and hypertension showed a positive dose–response relationship, with a pooled effect size of 1.21 (95% CI 0.78–1.87) for less than 80 dB(A), 1.77 (95% CI 1.36–2.29) for dB(A) between 80 and 85, and 3.50 (95% CI 1.56–7.86) for 85–≤90 dB(A), independently of the gender. [33] The first possible pathophysiological explanation of these results came from a study performed on animals ages ago. The activation of the sympathetic nervous system leads to an increase in catecholamines and then to vasoconstriction and high blood pressure [33]. Up to now, other pathophysiological mechanisms have been described. The endocrine system seems to also be important, with the increased release of cortisol and the enhancement of the activity of catecholamines [34]. The final result is the activation of the renin–angiotensin–aldosterone system. In addition, the mechanism mediating noise and blood pressure increase could begin from the activation of the amygdale nuclei and the sequent arterial inflammation [34].

The effect of noise on hypertension has been examined and proved to be both short- and long-term. In 2018, Shih-Yi Lu et al. investigated the adverse effects of high-frequency noise on BP in a group of thirty participants without a history of hypertension. During the study, participants were exposed to three distinct noise levels (<55, 75, and 90 dB) for 20 min, and their BP measurement as well as cardiac rhythm were recorded before and after the noise exposure. All participants successfully completed the task. As anticipated, different noise frequencies had varying impacts on BP levels. Notably, a significant increase in both systolic and diastolic BP was observed when participants were exposed to 90 dB noise. Contrasting the baseline noise level (<55 dB, pre-test) with higher noise levels, at 75 dB or 90 dB (post-test), participants showed heightened differences in both systolic and diastolic BP values. Specifically, the variance was more pronounced when subjects were exposed to louder noise, with systolic BP showing an increase of 3.9 ± 0.9 mmHg and diastolic BP increasing by 2.7 ± 0.7 mmHg at 90 dB. The analysis, using Student’s *t*-test, indicated a significant difference (*p* = 0.040) in diastolic BP levels at 75 dB, whereas there was no statistically significant difference observed in systolic BP. No difference was observed in cardiac rhythm. These findings imply that it is advisable to limit people’s exposure to sound levels no higher than 75 dB. This specific noise level exhibited the least impact on systolic BP [35].

The association between occupational noise and hypertension in workers in a local aircraft manufacturing enterprise in Xi’an, China was investigated in a cross-sectional study [36]. Results showed that exposure to high noise (>85 dB(A)) caused a 30% higher risk for hypertension compared to lower exposure, while in young adults, the odds were 70%. In accordance with this study, recent evidence from China shows that workers in Wuhan exposed to occupational noise >85 dB(A) had a higher rate of hypertension (21%), suggesting that more rigorous measures should be taken in the industries to ensure cardiovascular protection [37]. The dose–response relationship between noise and hypertension was confirmed also in automobile manufacturing workers [38].

Not only the working place but also the place of residence seems to be important. The relationship between road traffic noise and cardiovascular disease risk was examined with the UK Biobank, in a project involving the recruitment of more than 50,000 participants [36]. Road traffic noise exposure above 65 dB(A) was found to be enough to increase by 0.77% systolic and by 0.49% diastolic BP. This effect was prominent in highly exposed individuals, and it could be underestimated without the inclusion of the air pollution factor (NO2, PM2.5). According to the results of the DEBATS study in France [39], a 10 dB(A) increase in aircraft noise levels was associated with a 36% higher incidence of hypertension. A smaller study (114 male participants) [40] searched for the effect based on the duration of aircraft noise exposure in the systematic and renal circulation. In hypertensive but not in healthy individuals, 25 min of aircraft noise was enough to increase peripheral resistance and decrease the cardiac index, while renal circulation was not affected. Even when noise exposure occurs strictly during nighttime, a 5 dB(A) increase leads to higher levels of diastolic blood pressure, especially in people aged more than 65 years old and also when the noise is coming from a railway [41]. The increase in stress hormones and sequent oxidative stress may act as a possible mechanism explaining this observation [42].

The most crucial point is the long-term effect of noise on hypertension, as well as if this could be a possible answer to high rates of uncontrolled hypertension. A 4-year follow-up with more than 6000 participants revealed that an increase of 1.2 mmHg in systolic and 1.1 mmHg in diastolic blood pressure was associated with a 10 dB(A) higher noise level. Most importantly, this increase in noise was found to be associated with 20% increased odds of apparent treatment-resistant hypertension [43].

Beyond noise pollution, air pollution seems also to be an important environmental factor of hypertension [44]. Air pollution is a blend of particles, characterized by variations in their physical and chemical makeup, source, and harmful effects across different locations and times. Solid-phase air pollutants are typically categorized based on their size, with PM2.5 standing for particulate matter (PM) with a diameter of less than 2.5 μm, PM10 being particles with a diameter of less than 10 μm, PMcoarse encompassing particles between 2.5 and 10 μm in size, and ultrafine particles (UFP) indicating those with a diameter of less than 0.1 μm [45]. In studies focusing on short-term exposure, it was observed that increased levels of PM2.5 and PM10 were linked to elevated diastolic BP. Notably, only PM2.5 exhibited a similar association with increased systolic BP. When analyzing long-term exposures through meta-analysis, noteworthy associations were found between elevated diastolic BP and increased levels of PM2.5 and PM10. Furthermore, it is worth noting that there was a consistent association observed between extended exposure to PM2.5 and the development of incident hypertension [46,47,48,49,50]. Air pollution exerts its influence on the cardiovascular system by triggering inflammation and oxidation, which subsequently leads to heightened arterial stiffness and, consequently, the onset of hypertension. Moreover, an increase in BP can result from the activation of the sympathetic nervous system, which is triggered by airborne particles [51].

## 4. Hypertension and Socioeconomic Status

The importance of socioeconomic status, lifestyle preferences, and dietary practices should be researched extensively due to the close relationships between these factors and the onset, but also regulation, of hypertension. Mental health issues are also strongly associated with the above-mentioned factors and act as determinants of hypertension [52]. Conditions such as depression and anxiety disorders show a rapid increase nowadays and are affecting more than 300 million people worldwide [53]. Profound interconnections between these factors and hypertension are recognized. The activation of the sympathetic nervous system, the surge in cortisone, and the ongoing inflammation in stressful situations can cause hypertension through many different pathways [53,54,55]. These associations were researched in a recent study that assessed the responses provided by participants who had completed questionnaires regarding hypertension and disability. These questionnaires were administered during the 2017–2018 cycle of the National Health and Nutrition Examination Survey (NHANES). Individuals who were under treatment for depression and anxiety exhibited a higher likelihood of having clinical hypertension. Moreover, respondents who reported occasional experiences of depressive feelings demonstrated an increased likelihood of hypertension, even if these occurrences were infrequent. Similarly, participants who reported experiencing daily or weekly anxiety were also more prone to having hypertension [56].

In the sphere of socioeconomic status and lifestyle choices, the persistent issue of obesity is also rooted. Obesity is one of the most prominent risk factors for hypertension and both constitute major risk factors for cardiovascular disease [57,58]. The Framingham study was one of the first big data population studies to analyze in detail the relationship between hypertension and obesity, showing that a BMI ≥ 25 was responsible for 26% of the cases of hypertension in men and 28% in women [59]. Data from the United States show that an increase in BMI accounts for nearly all of the increases in hypertension in men, while some of the increases in hypertension in women were caused by factors other than BMI increase [60]. All these data are confirmed also from recently published, big data population studies [61,62,63]. Even if the relationship between obesity and hypertension is well studied and quite clear, still, there is space to investigate the environmental aspect as it functions between them, as the way we approach obesity and obesity-induced hypertension at the moment does not seem to work.

More and more households face the problem of food insecurity, defined either as limited availability of or inability to have nutritionally safe food [64]. In the United States, the overall age-standardized rate of food insecurity doubled from 2005 to 2012 [65]. The ramifications of food insecurity in health can be explained by the consumption of cheaper, unhealthy, more processed food, instead of healthier and more nutritional products that are more expensive [66]. If we consider that long-term consumption of fast food has already been correlated with BMI increase, in contrast to the Mediterranean diet, the economic environment seems to be of major importance for the increase in obesity and hypertension incidence [67,68]. Furthermore, a suboptimal diet has been associated with a 64% increase in all-cause cardiometabolic deaths in individuals aged 25–34 years, with the highest proportion of deaths associated with excess intake of sugar-sweetened beverages and processed meat [69]. The exposure to these products in childhood/adolescence led also to a 16% increase in hypertension development after a 17-year follow-up in subjects aged 9–16 years [70].

Also, the economic extenders of food insecurity can lead to individuals preferring to spend money on food rather than on medication (e.g., antihypertensive drugs) [71,72]. The psychological effect of food insecurity should not be ignored as it increases stress and causes anxiety and depression [73,74]. Furthermore, advertisement is all around, mostly promoting unhealthy food and almost never fruits or vegetables and creating behaviors. A meta-analysis showed that dietary intake in children significantly increased during or shortly after exposure to advertisements. Similarly, unhealthy dietary marketing caused a 10% higher risk of selecting the advertised foods or beverages [75]. All of the above can lead to a higher risk for hypertension and cardiovascular events.

Socioeconomic status affects hypertension also in many other different aspects. Residents of neighborhoods with poor conditions regarding walkability, availability of healthy food, safety, and social cohesion were more likely to have hypertension [76]. The insecurity regarding work may also affect both women and men and lead, long-term, to hypertension [77]. As such, income decreases and difficulty in paying even the basics have further been well associated with hypertension incidence, ref. [78] showing the importance of socioeconomic stress on this major cardiovascular risk factor.

## 5. Discussion

The aim of this review was to analyze how the environment we live in affects hypertension in an attempt to see beyond the obviously related causes of hypertension and to find new ways to stop the increasing hypertension incidence. Months of the year or even days of the week, exposure to high noise (especially higher than 85 dB(A)) either at work or at home, air pollution, obesity/unhealthy food as a result of social deprivation, media influence on food choices, neighborhood or work conditions, and stress all seem to be of major importance regarding hypertension.

In recent years, there has been an increasing discussion around the concept of the exposome. The multifactorial nature of the exposome necessitates targeted and extensive studies to comprehensively understand the social, mental, and environmental influences on hypertension and, by extension, cardiovascular disease [9]. As shown above, data clearly indicate the direction that most of the hypertension cases are not exactly "primary", but secondary, responses to the exposome. However, hypertension committees still do not include this important factor in their guidelines and do not alert clinicians or patients, but also governments.

The practical implication of this review is that there is a huge need for socioeconomic and environmental changes. Obviously, treating hypertension with medications should not be replaced, but these changes may act synergistically. Providing higher indoor temperatures during winter as well as lowering the noise at homes or workplaces could be some suggestions. At this point, more research is needed to investigate the optimal cut-off values. Furthermore, a balance between healthy and unhealthy food advertisements being controlled by governments, educational programs at school, strictly healthy snack choices in schools or workplace canteens, and higher financial support for those facing social deprivation also need promotion. Employers have also to find ways to lower the “Monday stress” leading to hypertension and find ways to make the new week at work attractive. Regarding research, long-term follow-up studies have to be performed to identify which are the best interventions in order to prevent the growing incidence of hypertension. In the future, it is of major importance to promote collaboration between clinicians and governments to make the socioeconomic and environmental effects on hypertension clear to the public, to organize communities and schools in order to prevent or manage this association, and to support research for possible solutions.

## 6. Conclusions

Most cases of hypertension are classified as essential. Environmental factors like socioeconomic conditions, climate change, and lifestyle choices influence hypertension. A revision in the categorization of hypertension could be important, as even in the case of essential, primary hypertension, the environment could be found to constitute it as secondary. In real clinical practice, the hypertensive population should be assessed and managed by—in addition to the common lifestyle modifications and pharmacological treatment—the identification of socioeconomic and environmental risk factors and their modifications in collaboration with community programs.

## Abbreviations

Cardiovascular disease: CVD, blood pressure: BP.
